# Risk factors of catheter- associated bloodstream infection: Systematic review and meta-analysis

**DOI:** 10.1371/journal.pone.0282290

**Published:** 2023-03-23

**Authors:** Elisabeth Lafuente Cabrero, Roser Terradas Robledo, Anna Civit Cuñado, Diana García Sardelli, Carlota Hidalgo López, Dolors Giro Formatger, Laia Lacueva Perez, Cristina Esquinas López, Avelina Tortosa Moreno

**Affiliations:** 1 Infusion and Vascular Access Nurse, Parc de Salut Mar, Barcelona, Spain; 2 IMIM (Hospital del Mar Medical Research Institute), Barcelona, Spain; 3 Department of Nursing Methodology, Quality and Research, Barcelona, Spain; 4 Infection control Program Nurse, Epidemiology and Evaluation Department, Parc de Salut Mar, Barcelona, Spain; 5 Coordinator Department of Nursing Methodology, Quality and Research, Parc de Salut Mar, Barcelona, Spain; 6 Department of Pneumology, Hospital Universitari Vall d’Hebron, Barcelona, Spain; 7 Public Health, Mental, Maternal and Child Health Nursing Department, Barcelona, Spain; 8 Faculty of Medicine and Health Sciences, University of Barcelona, Barcelona, Spain; 9 Department of Basic Nursing, Faculty of Medicine and Health Sciences, University of Barcelona, Spain; Istanbul University-Cerrahpasa, Cerrahpasa Medical Faculty, TURKEY

## Abstract

**Introduction:**

The prevalence of catheter-associated bloodstream infections (CLABSI) is high and is a severe health problem associated with an increase in mortality and elevated economic costs. There are discrepancies related to the risk factors of CLABSI since the results published are very heterogeneous and there is no synthesis in the description of all the predisposing factors.

**Objective:**

We aimed to perform a systematic review and meta-analysis to synthesize and establish the risk factors predisposing to CLABSI reported in the literature.

**Method:**

This is a systematic review of observational studies following the PRISMA recommendations. MEDLINE and CINAHL databases were searched for primary studies from 2007 to 2021. The protocol was registered in PROSPERO CRD42018083564.

**Results:**

A total of 654 studies were identified, 23 of which were included in this systematic review. The meta-analysis included 17 studies and 9 risk factors were analyzed (total parenteral nutrition (TPN), chemotherapy, monolumen and bilumen catheters, days of catheterization, immunosuppression, kidney disease and diabetes mellitus) due to the homogeneity of their definitions and measurements. The risk factors found to increase the probability of developing CLABSI were TPN, multilumen devices, chemotherapy treatment, immunosuppression and the number of days of catheterization. On the other hand, monolumen devices presented a lower likelihood of triggering this infection.

## 1. Introduction

The use of central venous catheters (CVCs) has increased in current medical practice and is widely used in hospitalized patients [1, 2]. Safe administration of different medications and use by nursing teams is ensured by advances in the technology of these devices and insertion techniques, among others. However, despite the multiple benefits, CVCs are also associated with (central line)-associated bloodstream infections (CLABSI) [3–6].

In the United States 80,000 episodes of CLABSI are diagnosed annually and are associated with increased mortality and elevated economic costs (39,000 US dollars per episode) [7]. Despite including CLABSI in the Bacteremia 0 program and in nosocomial infection surveillance programs in Catalonia (VINCAT) or the Study of the Prevalence of Nosocomial Infections in Spain (EPINE), the rates of CLABSI remain elevated in our country [8]. According to EPINE, 45.80% of nosocomial bacteremias are secondary to a vascular device, with central venous access devices and peripherally inserted central catheters (PICC) being the cause in 34.39% and 11.42% of the cases, respectively [1]. The Spanish Society of Infectious Diseases and Clinical Microbiology (SEIMC) reports that the rates of CLABSI range between 15% and 30% in Spain [3]. Other international studies have reported catheter-associated infection rates of 6.3% to 23% of all nosocomial bacteremias and others describe 15.2% [9, 10]. Moreover, the high prevalence of this complication has led to it becoming one of the major causes of morbidity and mortality in hospitalized patients [5, 11]. According to SEIMC, the direct mortality attributable to bacteremia is between 12% and 25% [3, 12], with a repercussion on the health care system of a mean cost of 18,000 euros per episode, depending on the causative microorganism [13].

In addition to the high rates and severity of outcomes, many studies have described a multitude of risk factors. In 2007, one systematic review studied the risk of CLABSI based on the venous device implanted and the time in place [14]. However, this study did not evaluate other related risk factors that could increase the risk of CLABSI, such as those related to some treatments [4, 5, 15, 16], pathological history [5, 17–21] and clinical status [5, 18, 20, 22]. Thus, the results obtained in the different studies are very heterogeneous, and do not synthesize and identify all the factors that favor the appearance of CLABSI. Therefore, here we provide a systematic review and meta-analysis that synthesizes and establishes the risk factors predisposing central venous catheter-associated bacteremia described in the literature.

## 2. Method

### 2.1 Design

In accordance with the prevailing guidelines, our systematic review protocol was registered with the International Prospective Register of Systematic Reviews (PROSPERO, registration number CRD42018083564). This systematic review followed the guidelines of the Preferred Reporting Items for Systematic Reviews and Meta-Analyses (PRISMA) [23].

### 2.2 Search strategy

We performed serial literature searches for articles published in MEDLINE (via PubMed) and CINAHL, from 2007 to February 25, 2021, using the following keywords: “CLABSI”, “CRBSI” “Catheter” and “Risk factor”. Boolean operators were used to enhance electronic searches. All human studies published in full-text form were eligible for inclusion, with no language restriction in the searches. Additional studies of interest were identified by hand searches of bibliographies of expert authors (Pittiruti, M and Maki, D) (S1 Text).

### 2.3 Study eligibility and selection criteria

Three authors (EL, AT and CE) independently determined study eligibility. Any difference in opinion regarding eligibility was resolved through consensus.

Studies were included if they: involved participants 18 years of age or older; mentioned the risk factors associated with central venous devices, whether centrally or peripherally inserted (CICC/PICC, respectively); definition of catheter- associated bacteremia according to the criteria of the Centers for Disease Control (CDC)/National Healthcare Safety Network (NHSN); studies published in the last 14 years; and the study design was randomized control trials, cohort or case-control studies. We excluded studies with patients not hospitalized during the whole study.

### 2.4 Definition of variables and outcomes

The primary outcome of this study was the presence of CLABSIs or (central line)-related bloodstream infection (CRBSI) in patients with CICC or PICC.

A CICC was defined as any central venous access device inserted into the internal jugular or subclavian vein. PICCs were defined as catheters inserted in the basilic, axillar, cephalic, or brachial veins of the upper extremities with tips terminating in the cavoatrial junction. CLABSI or CRBSI was defined as the occurrence of bacteremia in patients with PICCs or CICCs according to CDC /NHSN criteria [7]

### 2.5 Data abstraction and validity assessment

Data were extracted from the studies included with use of a standardized template designed by our group. The following information was collected from all studies: study characteristics (author, year of publication, country, study design and patient population), variables related to vascular access/device (vascular access device, CLABSI ratio), variables and potential risk factors evaluated in each study and results of multivariate analysis.

### 2.6 Study selection

All the studies containing abstracts and title were imported to Mendeley (version 1.19.3; Mendeley LDT, m Elsevier, London, United Kingdom). After excluding duplicate papers, three investigators (EL, AT and CE) independently screened the title and the abstract according to the inclusion and exclusion criteria. If the selection of the literature could be determined based on the criteria, the full text was further evaluated. Three investigators (EL, AT and CE) independently assessed the quality of the papers included. The grade of evidence and grade of recommendation were established according to the proposal of the Centre for Evidence-Based Medicine of Oxford [24]

### 2.7 Range of bias among the studies

The three authors (EL, AT and CE) independently evaluated the risk of bias.

To analyze the quality of potentially eligible articles the Strengthening the Reporting of Observational studies in Epidemiology (STROBE) [25] statement for cohort, case and control studies was followed.

### 2.8 Inclusion in the meta-analysis, data extraction and statistical methods

A meta-analysis was performed using the most prevalent risk factors for the presence of CLABSI included in the quantitative review (total parenteral nutrition [TPN], number of lumens, days of catheter placement, chemotherapy, immunosuppression, kidney disease and diabetes).

For the data analysis in the case of days of catheterization, mean values and their standard deviations of each study were extracted and weighted mean differences and 95% confidence intervals (CI) were used. In the case of qualitative factors, odds ratios (OR) and 95% CI were calculated for each study. The Cochrane-Q test was performed to assess the degree of heterogeneity among studies, and the I2 index (Higgins et al. 2003) [26] was used to describe the percentage of variation across studies due to heterogeneity (I2 = 25%: low; I2 = 50%: moderate; I2 = 75%: high heterogeneity). Study-specific estimates were pooled using both the fixed effect model (Mantel–Haenzel–Peto test) and the random effect model (Dersimonian-Laird test). If significant heterogeneity was found, the random effect model results were shown. To the contrary, the fixed-effect model was presented. Forest Plots were created to describe the pooled analysis. Statistical significance was defined as a *P* value **<** 0.05. All of the statistical analyses were conducted using R Studio.

## 3. Results

### 3.1 Search results

**Fig 1:**
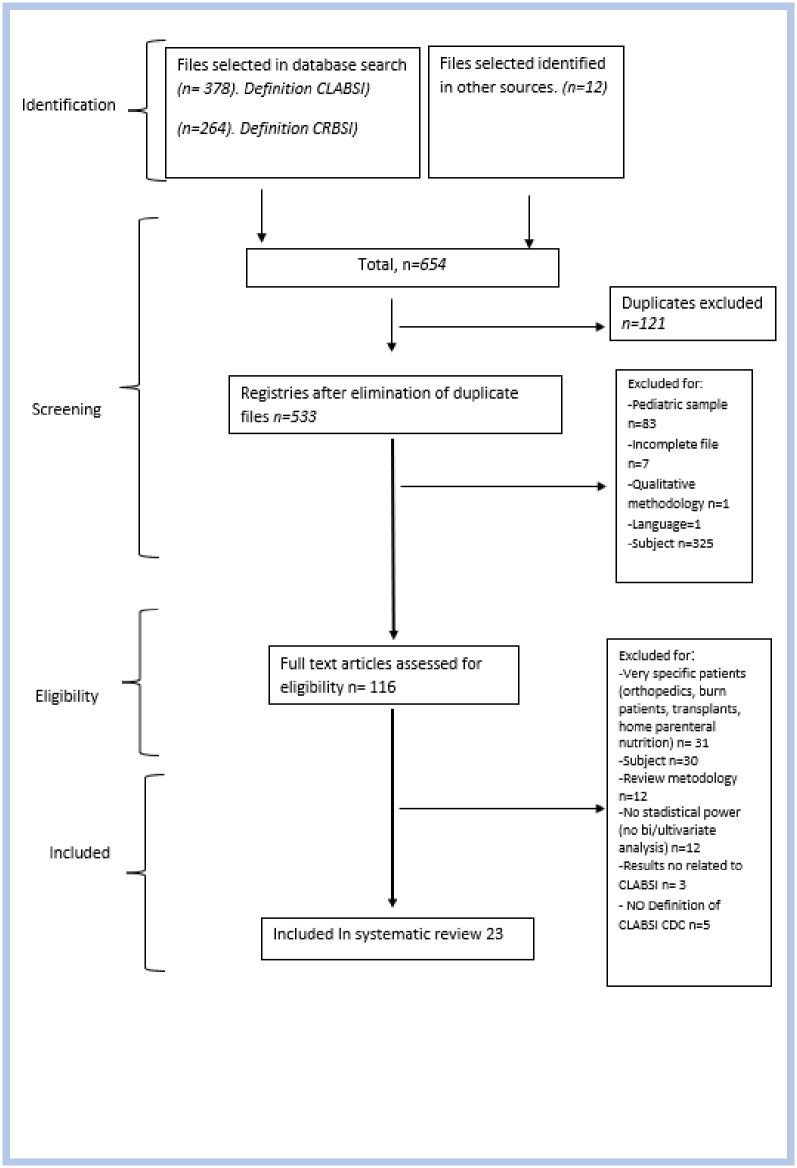
Flow diagram of study selection.

After removal of duplicates, 533 articles were identified by our electronic search. Of these, 417 were excluded on the basis of abstract information, and an additional 93 studies were discarded after full text review. Therefore, 23 studies reporting CLABSI in patients with PICCs or CICCs were included in the present systematic review. (Fig 1).

### 3.2 Characteristics of the studies included

Table 1 provides a detailed description of the studies analyzed. The 23 studies included were published between 2007 and 2021. Eight studies were undertaken in the United States, [4, 16,17, 20, 21, 27–29], three in Australia [30–32], two in India [18, 33], two in China [34, 35], and one in each of the following countries: Spain [36], Tunisia [19], Japan [15], France [37], Cyprus [38], Germany [22], Korea [39], and Turkey [40]. Among the studies eligible, 22 (95.65%) were cohort follow-up studies [4, 15, 17–22, 27–40] and 1 was a case-control study (4.34%) [16]. Of the studies included, 9 were performed in the Intensive Care Unit (ICU) (39.13%) [17–19, 27, 28, 30, 31, 33, 36], 10 in conventional hospitalization wards (43.47%) [4, 15, 16, 20, 21, 29, 35, 37, 38, 40] and 4 in the Oncology Department (17.39%) [22, 32, 34, 39].

**Table 1:** Characteristics of the studies included.

STUDY	COUNTRY	STUDY DESIGN	POPULATION	VASCULAR ACCESS DEVICE	CLABSI RATIO	CLABSI DEFINITON	RISK FACTOR	MULTIVARIATE	LEVEL OF EVIDENCE
Spelman et al 2017 [30]	Australia	Cohort follow- up	ICU patients	CICC	No information	CDC/NHSN	Age Any infectious diagnosis. Ventilation in the first 24 h. Policy of mandatory ultrasound guidance to localize CVC. Number of registered nurses Total hours receiving invasive ventilation Total hours receiving non-invasive ventilation Number of full-time specialists Number of specialists in session Total number of non-intensive care specialists Annual number of patients with known mortality. Years APACHE III score Number of invasive ventilations. Number of invasive ventilations. Number of non-invasively ventilated patients.	Patients with known mortality RR 1.11; 95% CI 1.04–1.19) P = 0.002 APACHE III (RR 1.03; 95%CI^b^ 1.01–1.06) P = 0.031 Total hours receiving invasive ventilation (RR 1.14; 95%CI^b^ 1. 08–1.21) P < 0. 001 Total hours with non-invasive ventilation per 100 days in bed (RR 1.01; 95%CI^b^ 1.01–1.02) P <0.001. Number of hours with non-invasive ventilation x 100 days in bed (RR 1.07; 95%CI 1.01–1.13) P = 0.030. Ultrasound guided device placement (RR 0.47; 95%CI 0.34–0.64) P < 0.001 Median age (RR 0.94; 95%CI 0.90–0.99) P = 0.02 Ventilation in first 24 hours (RR 0.85; 95%CI 0.77–0.94) P = 0.002	3a
Sarah S. Jackson et al 2017 [17]	Michigan USA	Cohort follow- up	ICU	CICC	85849/162	CDC/ NHSN	Age Sex Race ICU type Coagulopathy Dementia Uncomplicated diabetes Complicated diabetes Drug abuse Paralysis HIV Lymphoma Malignancy Metastatic cancer Liver disease Obesity Kidney disease Weight loss (malnutrition)	ICU medical/ surgical critical care (HR 1.83; 95%CI 1.04–3.20) P = 0.034 Coagulopathy (HR 1.65; 95% CI 1.17–2.30) P = 0.004 Paralysis (HR 1.76; 95%CI 1.06–2.93) P = 0.029 Kidney disease (HR 1.59; 95%CI 1.13–2.22) P = 0.007 Weight loss (HR 1.56; 95%CI 1.12–2.19) P = 0.01 Age per 10-year increase (HR 0.88; 95%CI 0.80–0.96) P = 0.006	3a
Kaur et al 2015 [33]	India	Cohort follow- up	ICU patients	CICC	90/25	CDC/NHSN	Age Gender Primary clinical diagnosis Catheter insertion site Multilumen catheter Duration catheterization Local sign of inflammation Length of ICU stay Death or survive Underlying comorbidity	Duration of catheterization (OR 9.83; 95%CI 1.21–80.05) P = 0.03 Erythema (OR 4.61; 95%CI 1.43–14.78) P = 0.012 Length of ICU stay >20 days (OR 4.80; 95%CI 1.69–13.62) P = 0.003	3b
Matew Lissauer et al 2011 [27]	Maryland USA	Cohort follow- up	ICU	CICC PICC	961/65	CDC/NHSN	Gender Source of ICU admission Readmission to SICU during current hospital admission Primary admission service Charlson comorbidity index National predicted ICU Mortality Reopening of recent laparotomy	Emergency surgery (OR 1.92 95%CI 1.02–3.61) National predicted ICU mortality: Quartile3 (OR 39.63; 95%CI 1.22–76.3) Quartile 4 (OR 20.8; 95%CI 2.7–162.3) Reopening of recent laparotomy (OR 2.08; 95%CI 1.10–3.94) Gender male (OR 1.93; 95%CI 1.02–3.68)	3a
Jose Garnacho–Montero 2008 [36]	Spain	Cohort follow- up	ICU	CICC PICC	1366/66	CDC/NHSN	Age APACHEII Gender Type of patients Comorbidities Site of catheter insertion Type of catheter Catheter insertion Number of lumens Material Antiseptic Use of three–way- stopcock Use to measure to CVP Concomitant Infection Duration of catheterization Catheter use Catheter insertion Site of insertion Number of lumens	Change over the guide (OR 4.59; 95%CI 2.28–9.3) P = 0.0001 Duration of catheterization (days) (OR 1.028; 95%CI 1.0009–1.048) P = 0.003 Tracheostomy (OR 2.3; 95%CI 1.17–4.54) P = 0.016	3a
S.W. Wong et al 2016 [31]	Australia	Cohort follow- up	ICU	CICC PICC	6307/46	CDC/NHSN	Age Sex Apache II/III Patient type Admission type Access site Catheter type Lumen ICU	Double-lumen catheter (OR 2.59; 95%CI 1.16–5.77) P = 0.02 Insertion before 2011 (OR 2.20; 95%CI 1.22–3.97) P < .0.01 ICU CVC-days > 7 (OR 2.07; 95%CI 1.06–4.04) P = 0.03	3a
C Pepin et al 2015 [28]	Maryland USA	Cohort follow- up	ICU	CICC	4011/76	CDC/NHSN	Chronic disease score, mean Charlson comorbidity index total Central-line days Age Sex	Days with (central line) (OR 1.04; 95%CI 1.03–1.06) P < .0001 Beta blocker and diuretic treatment (OR 1.85; 95%CI 1.04–3.29) P = 0.036 Kidney disease (OR 1.88; 95%CI 1.16–3.05) P = 0.010 Cholesterol lowering agents (OR 0.39; 95%CI 0.17–0.89) P = 0.026 Myocardial infarction (OR 0.28; 95%CI 0.10–0.76) P = 0.013	3a
SB Mishra et al 2016 [18]	India	Cohort follow- up	ICU	CICC	153/46	CDC/NHSN	Age Duration of hospitalization APACHE II SOFA Number of days with CVC Number of blood cultures sent Diabetes mellitus Hypertension COPD Coronary artery disease Immunosuppression Sepsis Pneumonia Intra-abdominal infection Bloodstream infection Mortality	Immunosuppression (OR 10.5; 95%CI 1.58–70.02) P = 0.015 Days with (central line) > 10 days (OR 5.52; 95%CI 1.8–16.1) P = 0.002	3b
Hajjej Z 2013 [19]	Tunisia	Cohort follow- up	ICU	CICC	482/54	CDC/NHSN	Age Sex APACHE II Reason for ICU admission Days in ICU Days with catheter Comorbidities Mechanical ventilation Sepsis at insertion One or more antibiotic Catheter site Parenteral nutrition Insertion context Mortality	Diabetes mellitus (OR 2.43; 95%CI 1.09–5.7) P0.027 Duration catheterization (OR 1.95; 95%CI 1.21–2.13) P<0.001 Sepsis at insertion (OR 3.80; 95%CI 1.91–7.87) P <0.001 ≥1 antibiotic before insertion (OR 4.46; 95%CI 2.08–10.1) P<0.001	3b
Ishizuka et al 2008 [4]	Japan		Hospitalized patients	PICC CICC	542/6	CDC/NHSN	Type of catheter Sex (male/female) Trouble with insertion Kinds of catheter Groshong catheter Argyle catheter 180 Types of disinfectant 10% povidone-iodine 0.05% chlorhexidine Administration of TPN Age Time catheter inserted Duration Type of chemotherapy	TPN (OR 12.75; 95%CI 2.48–62.26) P = 0.0023	3b
Herc et al 2017 [4]	Michigan USA	Cohort follow- up	Hospitalized patients	PICC	23088/249	CDC/NHSN	Race Age group BMI Pathological/surgical history TPN Hemodialysis Venous stasis Smoking status History of CLABSI Pharmacotherapy Analytical count Length of hospital stay prior to PICC placement CVC or PICC in prior 6 months Presence of another CVC Operator type Documented indication -PICC placement Hospital localization Arm selected Vein selected Device characteristics PICC gauge Type PICC	Hematological cancer (HR 3.77; 95%CI 2.75–5.16) P >0.001 CLABSI history within 3 months (HR 2.84; 95%CI 1.68–4.80) P >0.001 Active cancer with receipt of chemotherapy (HR 2.39; 95%CI 1.59–3.59) P >0.001 Multiple vs. single Lumen (HR 2.09; 95%CI 1.49–2.92) P>0.001 Presence of another CVC at time of PICC placement (HR 1.98; 95%CI 1.40–2.80) P>0.001 Receipt of TPN through the PICC (HR 1.82; 95%CI 1.21–2.73) P >0.001	3a
Sanjiv M et al 2013 [20]	Michigan USA	Cohort follow- up	Hospitalized patients	PICC	2193/57	—	Lumen Age Sex Diabetes Rheumatologic disease Immunosuppressed Recent chemotherapy PICC adjustment Power PICC PICC lumens	Immunosuppression (OR 2.60; 95%CI 1.45–4.67) P<0.01 3 PICC lumen compared with 1 lumen (OR 3.26; 95%CI 1.09–9.72) P = 0.02	3a
Caroline Bouzad et al 2015 [37]	France	Cohort follow- up	Hospitalized patients	PICC	923/31	CDC/NHSN	Gender Oncology disease Hematology ward Indication of PICC Placement chemotherapy Auto/allograft Other context Clamped PICC Senior operation High blood pressure Neutropenia Anti-coagulant therapy History of PICC/CVC Dwell time C/7hours/ 14 hours/21 hours	Chemotherapy (OR 7.2; 95%CI 1.8–29.6)P = 0.006 Auto/allograft (OR 6.0; 95%CI 1.2–29.3) P = 0.02 Anti-coagulant therapy (OR 4.1; 95%CI 1.4–12.0)P = 0.01	3a-3b
Makhawadee Pongruangporn et al 2013 [38]	Cyprus	Cohort follow- up	Hospitalized patients	PICC	485/162	CDC/NHSN	Demographic Comorbidity PICC description PICC where placed PICC insertion site Vein insertion Type of PICC	Congestive heart (OR 2.0; 95%CI 1.26–3.17) P = 0.003 Intraabdominal perforation (OR 5.66; 95%CI 1.76–18.19) P = 0.004 *Clostridium difficile* (OR 2.25; 95%CI 1.17–4.33) P = 0.02 Recent chemotherapy (OR 3.36; 95%CI 1.15–9.78) P = 0.03 Tracheostomy (OR 5.88; 95%CI 2.99–11.55) P<0.001 Double lumen (OR 1.89; 95%CI 1.15–3.10) P = 0.01 Trilumen (OR 2.87; 95% CI 1.39–5.92) P<0.001 Underlying COPD (OR 0.48; 95%CI 0.29–0.78) P = 0.03 Admission to surgical (OR 0.43; 95%CI 0.24–0.79) P = 0.006 Oncology and orthopedic (OR 0.35; 95%CI 0.13–0.99) P = 0.05	3a
P Ippolito et al 2015 [21]	New York USA	Cohort follow- up	Hospitalized patients	CICC	4840/220	CDC/NHSN	Age Charlson comorbidity Score Duration of parenteral nutrition Duration of catheterization Sex Underlying disease, Malignancy Diabetes mellitus HIV Kidney disease Surgical site infection, TPN History of transplant ICU stay Immunodeficiency Pneumonia	TPN (OR 4.33; 95%CI 2.50–7.48) P<0.001 Kidney disease (OR 2.79; 95%CI 2.00–3.88) P<0.001 ICU stay (OR 2.26; 95%CI 1.58–3.23) P<0.001 Immunodeficiency (OR 2.26; 95%CI 1.70–3.00) P<0.001 Diabetes (OR 0.63; 95%CI 0.45–0.88) P = 0.007	3a
V Chopra et al 2014 [29]	Michigan USA	Cohort follow- up	Hospitalized patients	CICC PICC	908/58	CDC/NHSN	Age Sex Admitting Ward Comorbidities Markers of severe illness PICC characteristics Primary indication for PICC -Insertion Arm of PICC insertion Vein of PICC insertion PICC insertion unit/ward PICC operator/inserter Number of PICC lumens PICC gauge/thickness (French)	Hospital length of stay (HR 1.02; 95%CI 1.00–1.04) P = 0 .003 Intensive care unit status (HR 1.02; 95%CI 1.01–1.02) P<0.0001 Number lumen 2 (HR 4.08; 95%CI 1.51–11.02) P = 0.006 Number lumen 3 (HR 8.52; 95%CI 2.55–28.49) P = 0.0003	3a
C Conccanon et al 2014 [16]	New York USA	Case/control	Hospitalized patients	CICC PICC	207/197	CDC/NHSN	Multiple CVC Sex TPN Hemodialysis Chemotherapy ICU Stay Length of stay Age Charlson comorbidity APACHE II (Central line)–days	Multiple CVC (OR 3.4; 95%CI 2.2–8.4) TPN (OR 2.2; 95%CI 1.2–4.0) Chemotherapy (OR 8.2; 95%CI 3.4–19.9) Length of stay: 11–18 days (OR 5.8; 95%CI 2.8–12.3) 19–35 days (OR 6.5; 95%CI 3.0–3.7) >35 days (OR 6.5; 95%CI 3.0–14.0)	3b
Bekçibaçi et al 2019 [40]	Turkey	Follow-up of one cohort	Hospitalized patients	CICC	310/46	CDC/NHSN	Advanced age Hemodialysis Blood product infusion Total parenteral nutrition Catheter types: Double lumen Triple lumen Catheter location Subclavian vein Jugular vein Femoral vein Experience of applier Emergency indication for catheter insertion Asepsis compliance Kidney disease Hematologic problems Monitoring in ICU Diabetes mellitus Charlson comorbidity index score ≥5 Surgical intervention -Antibiotic treatment during catheterization Glycopeptide use	Advanced age (OR 1.02; 95%CI 1.00–1.04) P = 0.018 Duration of catheterization (OR 1.03; 95%CI 1.00–1.06) P = 0.010	3a
Shenghai Wu et al 2017 [35]	China	Follow-up of one cohort	Hospitalized patients	CICC	477/38	CDC/NHSN	Sex Primary disease Gastric cancer Colorectal cancer Rectal cancer -Gastrointestinal perforation Intestinal obstruction Peritonitis Surgical procedure Diabetes mellitus CVC days	Surgical procedure (OR 3.96; 95% CI 1.01–15.51)P = 0.05 CVC days (OR 1.08; 95% CI 1.04–1.13) P<0.001	3b
P. Mollee et al 2011 [32]	Australia	Cohort follow- up	Oncology patients	PICC CICC	1127/129	CDC/NHSN	Gender Nº of prior lines Neutrofilos Type line Side of the insertion Lumens Insertion site Diagnosis of patients Purpose of line Reason removal	Tunneled (HR 2.78; 95% CI 1.40–5.22) P = 0.0035 Non tunneled (HR 8.69; 95% CI 3.52–21.5) P< 0.0001 Aggressive hematological (HR 3.07; 95% CI 1.18–8.03) P = 0.022	3a
Yufang Gao et al 2015 [34]	China	Cohort follow- up	Oncology patients	CICC PICC	912/94	CDC/NHSN	Gender Age Underlying cancer Season of catheter placement Tumor type Placement time Insertion vein Insertion arm Insertion unit PICC adjustments PICC dislodgment Tip position Fixing method Catheter brand	Catheter care delay (OR 2.612; 95% CI 1.373–4.969) P = 0.003 Summer (OR 4.78; 95% CI 2.681–8.538) P<0.001 Tip position located in the lower third of the superior vena cava (OR 0.34; 95% CI 0.202–0.517) P<0.001 Statclok fixing (OR 0.55; 95% CI 0.326–0.945) P = 0.03	3a
Baier et al 2019 [22]	Germany	Follow-up of one cohort	Oncology patients	CICC PICC	610/111	CDC/NHSN	Age *>*50 years Acute myeloid leukemia Cardiac disease (comorbidity) Body mass index *>*30 kg/m2 Carbapenem therapy Aminoglycoside therapy Hematopoietic stem cell transplantation Allogenic hematopoietic stem cell/bone marrow transplantation Leukocytopenia *<*1,000/μL Anemia Thrombocytopenia *>*1 CVC inserted CVC insertion for conditioning phase Jugular vein insertion as CVC insertion site Non Hodgkin Lymphoma Transfusion of erythrocytes Subclavian vein as CVC insertion site Length of CVC use *<*8 days	Leukocytopenia *<*1,000/μL (OR 69.77 95% CI 15.76–308.86) P<0.001 *>*1 CVC inserted (OR 7.08; 95% CI 2.95–17) P<0.001 Carbapenem therapy inserted (OR 6.02; 95% CI 2.29–15.83) P<0.001 Pulmonary diseases (OR 3.17; 95% CI 1.32–7.62) P<0.001 Acute myeloid leukemia (OR 2.72; 95% CI 1.43–5.17) P = 0.002 CVC insertion for conditioning phase (OR 2.07; 95% CI 1.04–4.1) P = 0.037 Transfusion of erythrocytes (OR 0.04; 95% CI 0.02–0.08) P<0.001 Glycopeptide therapy (OR 0.10 95%; CI 0.03–0.34) P<0.001 Subclavian vein as CVC insertion site (OR 0.32; 95% CI 0.14–0.77) P = 0.010	3a
Lee et al 2020 [39]	Korea	Follow-up of one cohort	Oncology patients	PICC	539/25	CDC/NSHN	Mean age Sex History of ICU stay Presence of an additional intravascular device Hospital length of stay -Intravenous infusion TPN Antibiotic therapy Chemotherapy Catheter in place more than 3 weeks Single lumen Double lumen Right arm Left arm Basilic vein Brachial vein	Antibiotic therapy (HR 2.85; 95% CI 1.082–7.530) P = 0.034 Chemotherapy (HR 11.42; 95% CI 2.434–53.594) P = 0.002 Lumen (Single/Double) (HR 5.46; 95% CI 1.257–23.773) P = 0.024	3a-3b

OR, Odds Ratio; RR, Relative Risk; HR, Hazard Ratio; CI, Confidence Interval; CICC, central venous catheter central insertion; PICC, Peripheral Insertion Central Catheter; CDC, Center for Disease Control and Prevention; NHSN, National Healthcare Safety Network; APACHE, Acute Physiology and Chronic Health disease Classification System; ICU, Intensive Care Unit; HIV, human immunodeficiency virus; CVP, Central Venous Pressure; SOFA, Sepsis related Organ Failure Assessment; TPN, Total Parenteral Nutrition; BMI, Body Mass Index; SICU: surgical intensive care unit; COPD: chronic obstructive pulmonary disease; CVC, Central Venous Catheter; CLABSI, (Central-line) bloodstream infection.

All the studies specified the type of catheter used; in 9 the type of venous device used was CICC (39.13%) [17–19, 21, 28, 30, 33, 35, 40], in 5 PICC (21.73%) [4, 20, 37–39], and in 9 studies both types of devices were included (39.13%) [15, 16, 22, 27, 29, 31, 32, 34, 36].

The sample size of the studies evaluated established the catheter as the unit of analysis. In the cohort follow-up studies, the sample size ranged between 115 and 85,849 catheters, except in one study [30], which did not report the number of catheters but described rates of days of catheter placement. The only case-control study evaluated [16] included a sample of 197 cases and 207 controls.

### 3.3 Quality of the studies included

Analysis of the quality of the studies included was performed according to the STROBE statement [25]. The quality of the studies included was 3a and 3b. Eighteen studies obtained a grade 3a recommendation (78.26%) while 6 were 3b (21.74%). Of the latter 6 studies, one had a case-control design [16] and the 5 remaining studies [18, 19, 33, 35, 39] had a reduced sample size and did not achieve sufficient statistical power. Thus, the quality of the studies included in the review was good-regular.

### 3.4 Description of the risk factors

#### 3.4.1 Demographic characteristics

Gender was analyzed in 20 articles (89.95%), although male sex was identified as having a greater probability of CLABSI in only 1 study [27] (odds ratio [OR] 1.93; 95% confidence interval [CI] 1.02–3.68). Age was evaluated as a risk factor in 18 studies (78.26%). One study independently related age to the risk of CLABSI (OR 1.02; 95% CI 1.00–1.04) [50]. On the other hand, another study [30] demonstrated that age was a protective factor for CLABSI (relative risk [RR] = 0.94; 95% CI 0.90–0.99).

#### 3.4.2 Pharmacotherapy administered

Nine (39.13%) articles included the type of pharmacotherapy administered through both an inserted catheter and other administration routes as a study variable. In regard to the treatment administered through the endovenous device, one study related preventive administration of antibiotics prior to catheter insertion to the appearance of infection (OR 4.46; 95% CI 2.08–10.1) [19]. Another study related the administration of antibiotics through the endovenous device to the risk of infection (hazard ratio [HR] 2.854; 95% CI 1.082–7.530) [39]. Specifically, the administration of other drugs, such as carbapenems, was shown to be a risk factor for CRSBI (OR 6.02; 95% CI 2.29–15.83) [22]. To the contrary, the administration of glycopeptides and blood transfusions reduced the probability of catheter-associated infection (OR 0.10; 95% CI 0.03–0.34) and (OR 0.04; 95%CI 0.02–0.08), respectively [22]. The administration of chemotherapy was identified as a risk factor in different studies [4, 16, 37–39] (HR 2.39; 95% CI 1.59–3.59), (OR 7.2; 95% CI 1.8–29.6), (OR 3.36; 95%CI 1.15–9.78), (OR 8.2; 95% CI 3.4–19.9), (HR 11.421; 95% CI 2.434–53.594), respectively. Likewise, TPN was also shown to be a factor related to CLABSI in 4 articles [4, 15, 16, 21] (HR 1.82; 95% CI 1.21–2.73), (OR 12.75; 95% CI 2.48–62.26), (OR 4.33; 95% CI 2.50–7.48), (OR 2.2; 95% CI 1.2–4.0), respectively. Other factors related to CLABSI [28,37] were the administration of anticoagulants, beta-blockers and diuretics (OR 4.1; 95% CI 1.4–12.0) and (OR 1.85; 95% CI 1.04–3.29), respectively. Finally, cholesterol-reducing drugs (oral statins) were described as protective factors (OR 0.39; 95% CI 0.17–0.89) (28).

#### 3.4.3 Interventions and care in critical patients

One of the studies related ICU stay greater than 20 days as a factor which increased the probability of CLABSI (OR 4.80; 95% CI 1.69–13.62) [33]. Another article described the relation which both invasive mechanical ventilation (IMV) and non-invasive mechanical ventilation have with CLABSI (RR 1.14; 95% CI 1. 08–1.21) and (RR 1.01; 95% CI 1.01–1.02), respectively [30]. However, in the same study, IMV during the first 24 hours reduced the probability of developing CLABSI (RR 0.85; 95% CI 0.77–0.94). Only one study identified tracheostomy as a risk factor for CLABSI (OR 2.3; 95% CI 1.17–4.54) [36]. In the critical surgical setting, two studies reported that emergency surgery by laparotomy increased the probability of presenting CLABSI (OR 1.92; CI 95% 1.02–3.61) [27] and (OR 3.96; 95% CI 1.01–15.51) [35], and reopening was also considered a risk factor (OR 2.08; 95% CI 1.10–3.94) [27].

#### 3.4.4 Analytical indicators

Four studies evaluated the presence of immunological factors related to the risk of CLABSI, with two studies [18, 20] identifying immunosuppression as a risk factor (OR 10.5; 95% CI 1.58–70.02) and (OR 2.60; 95% CI 1.45–4.67), respectively. A third study related immunodeficiency to the appearance of CLABSI (OR 2.26; 95% CI 1.70–3.00) [21].

Autologous/allogenic hematopoietic stem cell transplantation showed a relationship with catheter-related infection (OR 6.0; 95% CI 1.2–29.3) [37]. Likewise, leucopenia also demonstrated a relationship with CLABSI (OR 69.77; 95% CI 15.76–308.86) [22].

On the other hand, three studies [4, 33, 38] reported that the presence of some microorganisms in different contexts increased the likelihood of developing CLABSI. Colonization-infection by *Clostridium difficile* (OR 2.25; 95% CI 1.17–4.33) [38], a history of CLABSI during the three months prior to new device placement (HR 2.84; 95% CI 1.68–4.80) [4] and sepsis of the exit-site (OR 4.61; 95% CI 1.43–14.78) and (OR 3.80; 95% CI 1.91–7.87) [19, 33] were independently related to CLABSI.

#### 3.4.5 Comorbidities

A higher score in the Acute Physiology and Chronic Health Disease Classification System (APACHE III) scale increased the probability of catheter-related sepsis (RR 1.03; 95% CI 1.01–1.06) [30], and coagulopathy was independently related to the appearance of CLABSI (HR 1.65; 95% CI 1.17–2.30) [17]. In addition, in the latter study other factors related to infection were identified: paralysis of the extremity carrying the device (HR 1.76; 95% CI 1.06–2.93) and weight loss (HR 1.56; 95% CI 1.12–2.19). Acute myocardial infarction was also found to be related to CLABSI (OR 0.28; 95% CI 0.1–0.76) [28].

Kidney disease was independently related to CRSBI in three studies (HR 1.59; 95% CI 1.13–2.22) [17], (OR 1.88; 95% CI 1.16–3.05) [28] and (OR 2.79; 95% CI 2.00–3.88) [21]. Pulmonary disease and acute myeloid leukemia were also related to the appearance of CLABSI in one study (OR 3.17; 95% CI 1.32–7.62) (OR 2.72; 95% CI 1.43–5.17), respectively [22]. In addition, two publications identified the presence of hematologic neoplastic disease as a risk factor (HR 3.07; 95% CI 1.18–8.03) [32] and (HR 3.77; 95% CI 2.75–5.16) [4]. In the case of diabetes, on one hand, in one study it was described as a risk factor (OR 2.43; 95% CI 1.09–5.7) [19] while in another study diabetes had a protector effect (OR 0.63; 95% CI 0.45–0.88) [21].

#### 3.4.6 Catheter

With regard to catheter-related variables, one study showed that replacing the catheter through a guideline increased the probability of developing catheter infection (OR 4.59; 95% CI 2.28–9.3) [36]. The number of lumens was also related to the appearance of CLABSI in five studies [4, 20, 29, 38, 39], showing that the greater the number of lumens the greater the likelihood of developing infection (HR 2.09; 95% CI 1.49–2.92), (OR 3.26; 95% CI 1.09–9.72), (OR 2.87; 95% CI 1.39–5.92) (HR 8.52; 95% CI 2.55–28.49), and (HR 5.466; 95% CI 1.257–23.773), respectively. The synchronic presence of other venous devices also influenced the appearance of infection (HR 1.98; 95% CI 1.40–2.80) [4] and (OR 3.4; 95% CI 1.7–6.9) [16], (OR 7.08; 95% CI 2.95–17) [22]. On the other hand, the latter study also demonstrated that insertion into the subclavian vein had a protective effect (OR 0.32; 95% CI 0.14–0.77) [22]. Other protective factors reported included ultrasound-guided insertion (RR 0.47; 95% CI 0.34–0.64) [30], correct positioning of the distal point in the lower third of the superior vena cava (OR 0.34; 95% CI 0.2–0.51) [34] and an adhesive fixation system (OR 0.55; 95% CI 0.32–0.94) [34].

#### 3.4.7 Temporality

According to the results of four studies, the duration of device implantation had an impact on the appearance of catheter-related bacteremia, being one of the variables most frequently studied and showing the greatest number of significant results (OR 1.028; 95% CI 1.0009–1.048) [36], (OR 1.04; 95% CI 1.03–1.06) [28], (OR 5.52; 95% CI 1.8–16.1) [18], (OR 1.95; 95% CI 1.21–2.13) [19], (OR 1.08; 95% CI 1.04–1.13) [35], (OR 1.02; 95% CI 1.00–1.04) [40]. Two studies related the length of ICU stay to the appearance of CLABSI, with one showing that a stay longer than 7 days increased the probability of the infection and the second determined that a stay greater than 20 days was a factor related to infection (OR 4.80; 95% CI 1.69–13.62) [33] and (OR 2.07; 95% CI 1.06–4.04) [31].

#### 3.4.8 Microbiology

Microbiological results were reported in 14 (60.8%) of the studies included in this systematic review. In 11 studies Gram-positive microorganisms were isolated: in 9 studies [20, 22, 29, 32,34–36, 38, 40] coagulase-negative Staphylococci were described as the most prevalent, with 4 identifying *S*. *epidermidis* [22, 32, 34, 38]. In another study, the most prevalent microorganism was *S*. *aureus* [33] and lastly, *Enterobacter* spp. [31]. In 4 studies [18, 19, 31, 39] Gram-negative bacilli were described as the most prevalent (*Enterobacter* spp., *Escherichia coli*, *Klebsiella pneumoniae*, *Pseudomonas aeruginosa*). Finally, *Candida* spp. was also isolated [31, 35, 39]. Some studies were cited twice because both microorganisms were isolated with the same prevalence.

### 3.5 Synthesis of the results

Among the 23 studies included, 17 were included in the meta-analysis [4, 15, 16–21, 28, 29, 33, 35–40]. The reasons for excluding six articles were: 1) the remaining risk factors were not defined or measured in the same way and did not allow for conclusive statistical tests, 2) they had not been analyzed in more than one study, and 3) the results of the studies did not show significance in the analyses performed. A total of 9 risk factors were identified and included in the meta-analysis due to the homogeneity of the definitions and measurements: administration of TPN, single, bilumen, or multilumen catheters (including trilumen, tetralumen and pentalumen catheters in the latter group), days of catheterization, chemotherapy, immunosuppression, kidney disease and diabetes mellitus.

**Fig 2:**
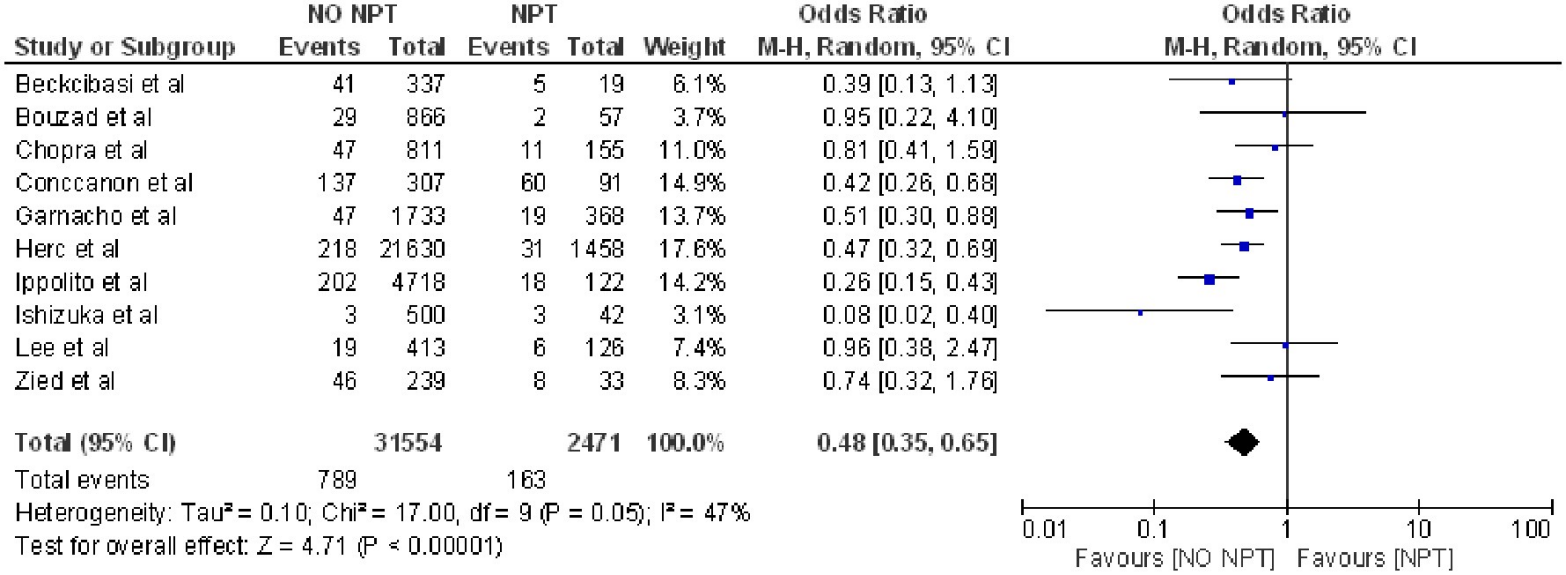
Forest plot of total parenteral nutrition (TPN) and CLABSI.

The results showed that patients not receiving TPN had a lower probability of having CLABSI (OR = 0.48; 95% CI: 0.35–0.65, p <0.001, heterogeneity I^2^ = 47%) [4, 15, 16, 19, 21, 29, 36, 37, 39, 40] (Fig 2).

**Fig 3:**
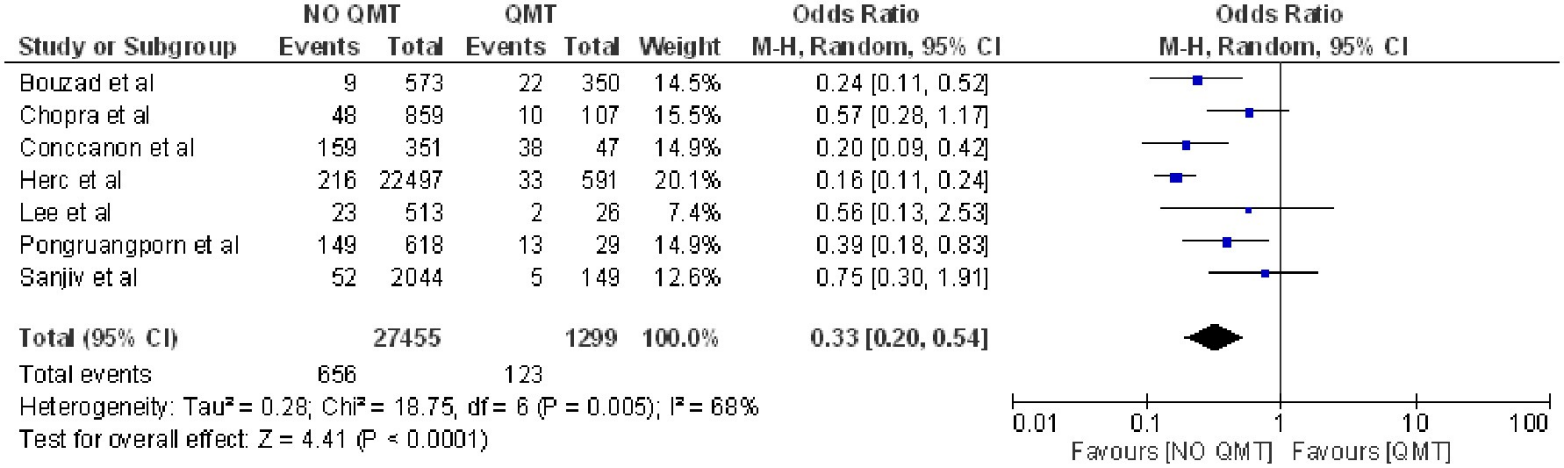
Forest plot of chemotherapy treatment and CLABSI.

Likewise, patients who did not undergo chemotherapy presented a lower probability of developing this complication (OR 0.33; 95% CI: 0.20–0.54, p<0.0001 heterogeneity I^2^ = 68%) [4, 16, 20, 29, 37–39] (Fig 3).

**Fig 4:**
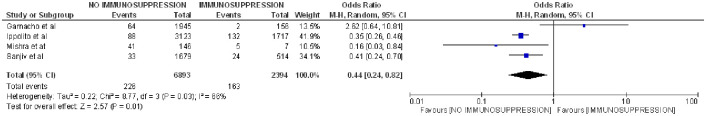
Forest Plot of immune system ompromiso and CLABSI.

Absence of immune system compromise secondary to treatment or some type of disease was also related to being a protector factor against CLABSI (OR 0.44; 95% CI: 0.24–0.82, p = 0.01, heterogeneity I^2^ = 66%) [18, 20, 21, 36] (Fig 4).

**Fig 5:**
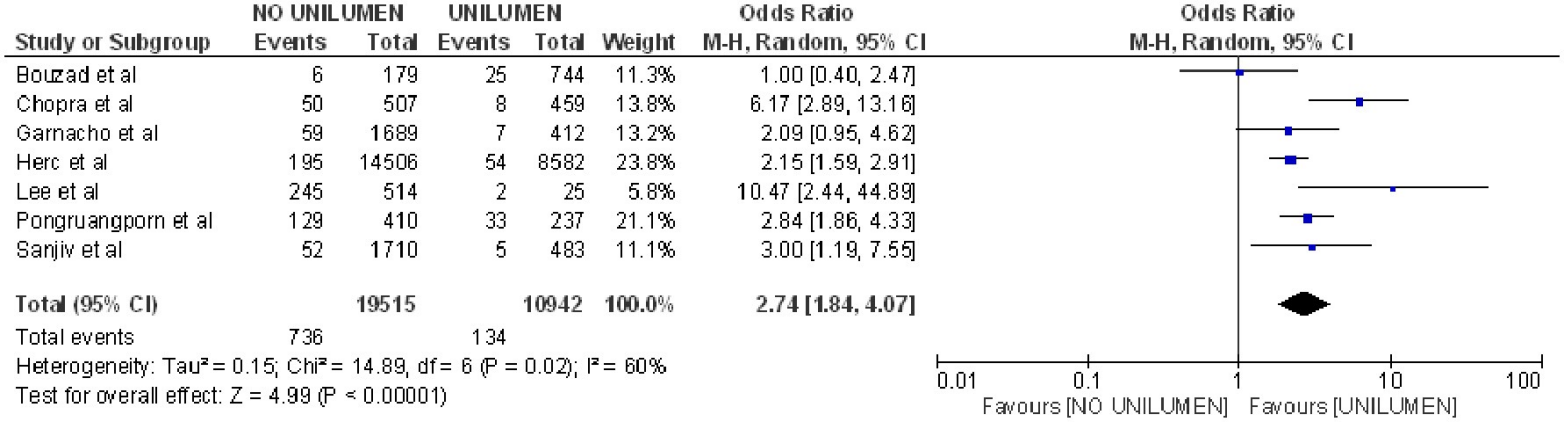
Forest Plot of unilumen catheter and CLABSI.

Being a carrier of a CVC with more than one lumen implied a greater risk of CLABSI (OR = 2.74; 95% CI: 1.84–4.07, p = 0.02, heterogeneity I^2^ = 60%) [4, 20, 29, 36–39] (Fig 5).

**Fig 6:**
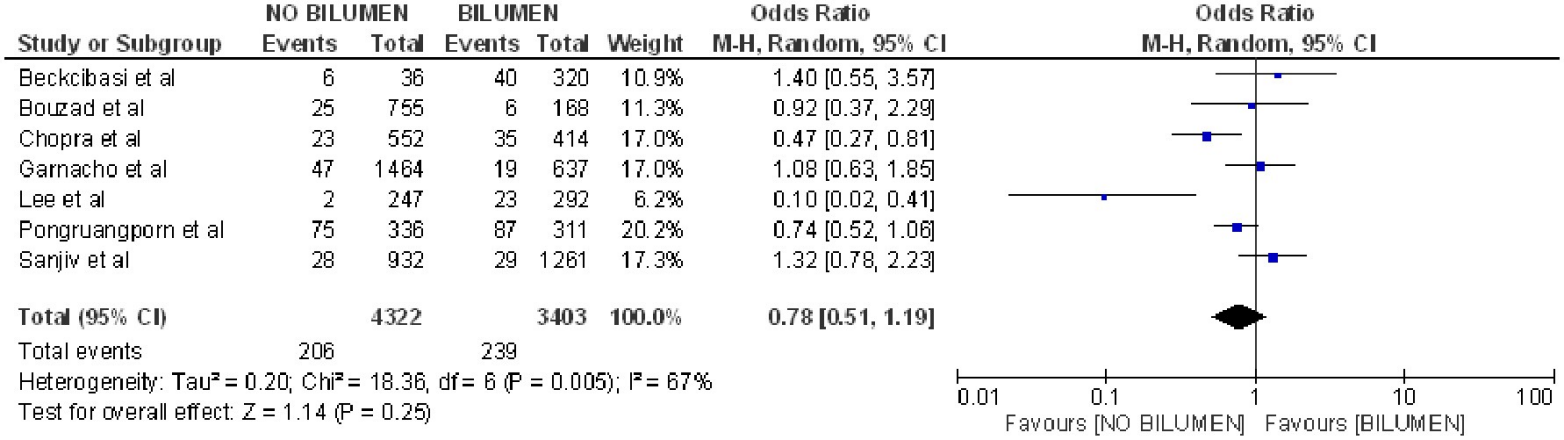
Forest plot of bilumen catheter and CLABSI.

On the other hand, bilumen devices analyzed in 7 articles [20, 29, 36–40] were not related to the appearance of CLABSI (OR 0.78; 95% CI: 0.51–1.19, p = 0.25, heterogeneity I^2^ = 67%) (Fig 6).

**Fig 7:**
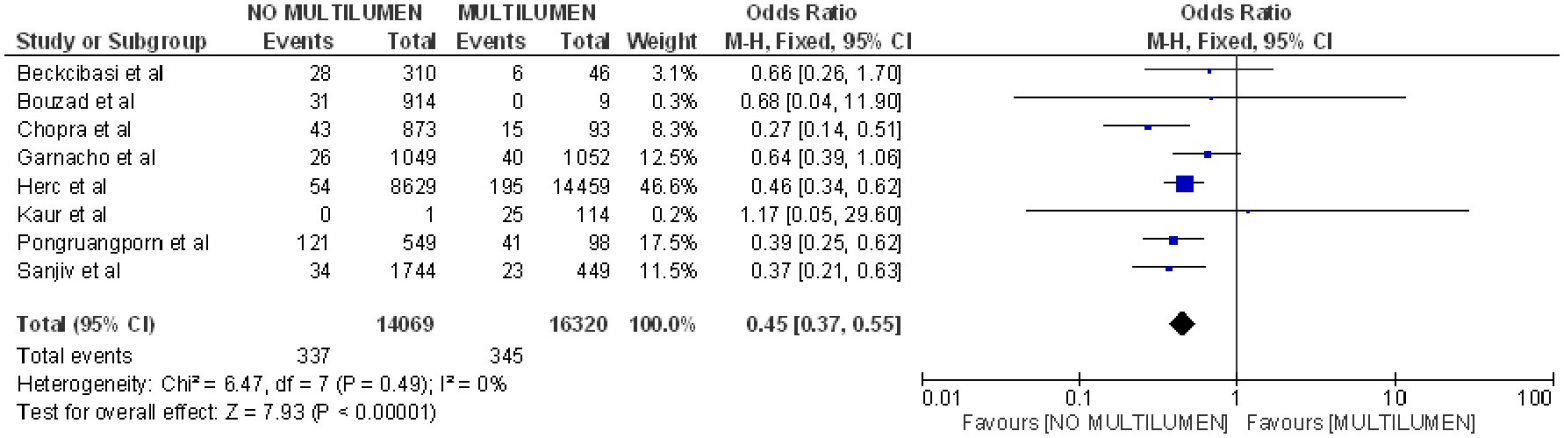
Forest plot of multilumen catheter and CLABSI.

Lastly, it was observed that not having a multilumen catheter reduced the probability of CLABSI (OR 0.45; 95% CI: 0.37–0.55, p<0.001, heterogeneity I^2^ = 0%) [(4, 20, 29, 33, 36–38, 40] (Fig 7).

**Fig 8:**
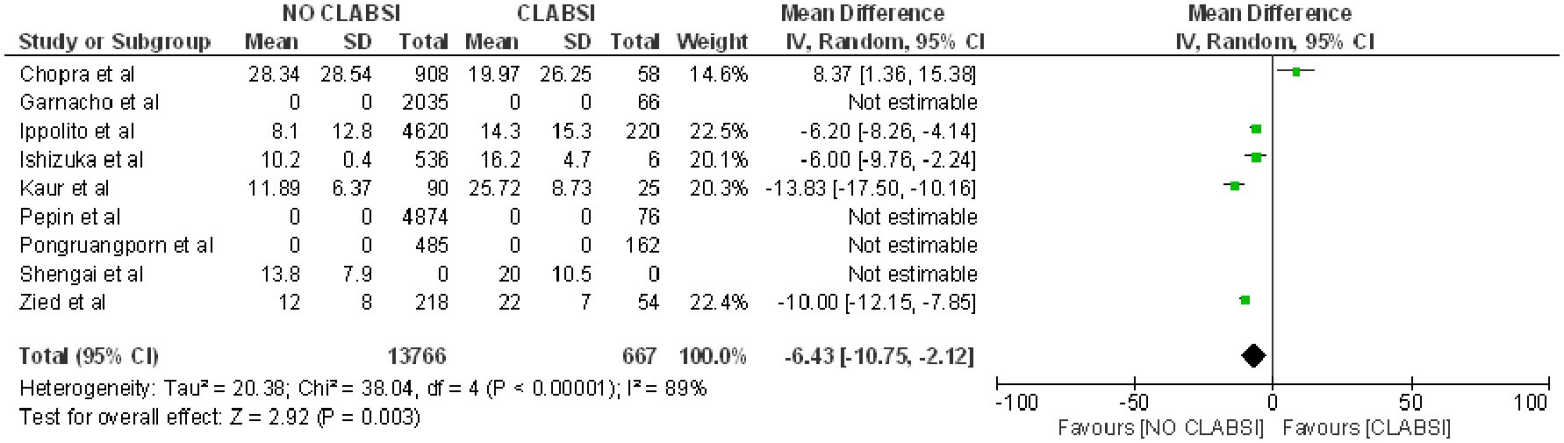
Forest plot of catheter days and CLABSI.

With regard to the number of days with a catheter, it was found that patients catheterized for a greater number of days had a higher likelihood of developing CLABSI (OR 6.43; 95% CI: 10.75–2.12, p = 0.003, heterogeneity I^2^ = 89%) [15, 19, 21, 29] (Fig 8).

**Fig 9:**
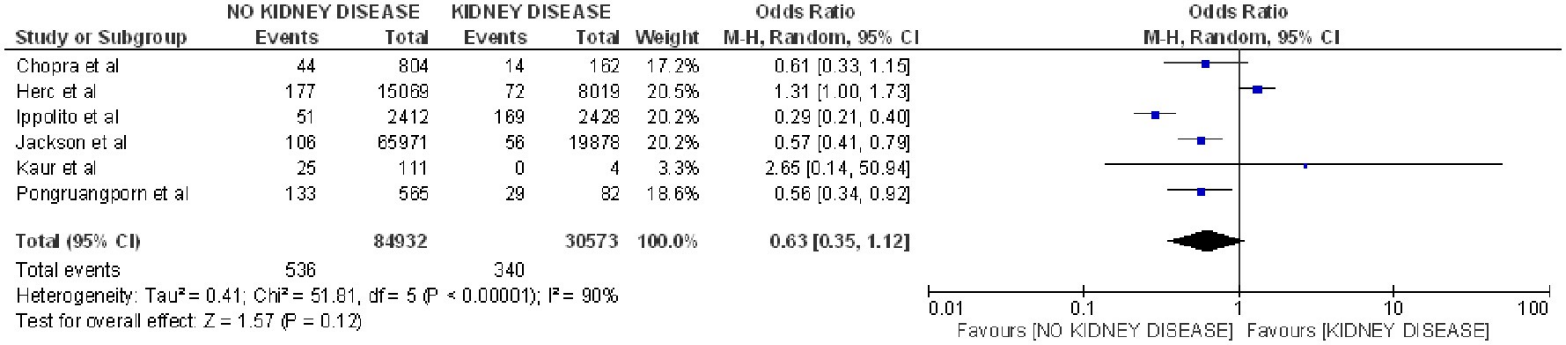
Forest plot of kidney disease and CLABSI.

Lastly, kidney disease was included in a total of 6 articles [4, 17, 21, 29, 33, 38] and showed no relationship with CLABSI (OR 0.63; 95% CI: 0.35–1.12, p = 0.12, heterogeneity I^2^ = 90%) (Fig 9).

**Fig 10:**
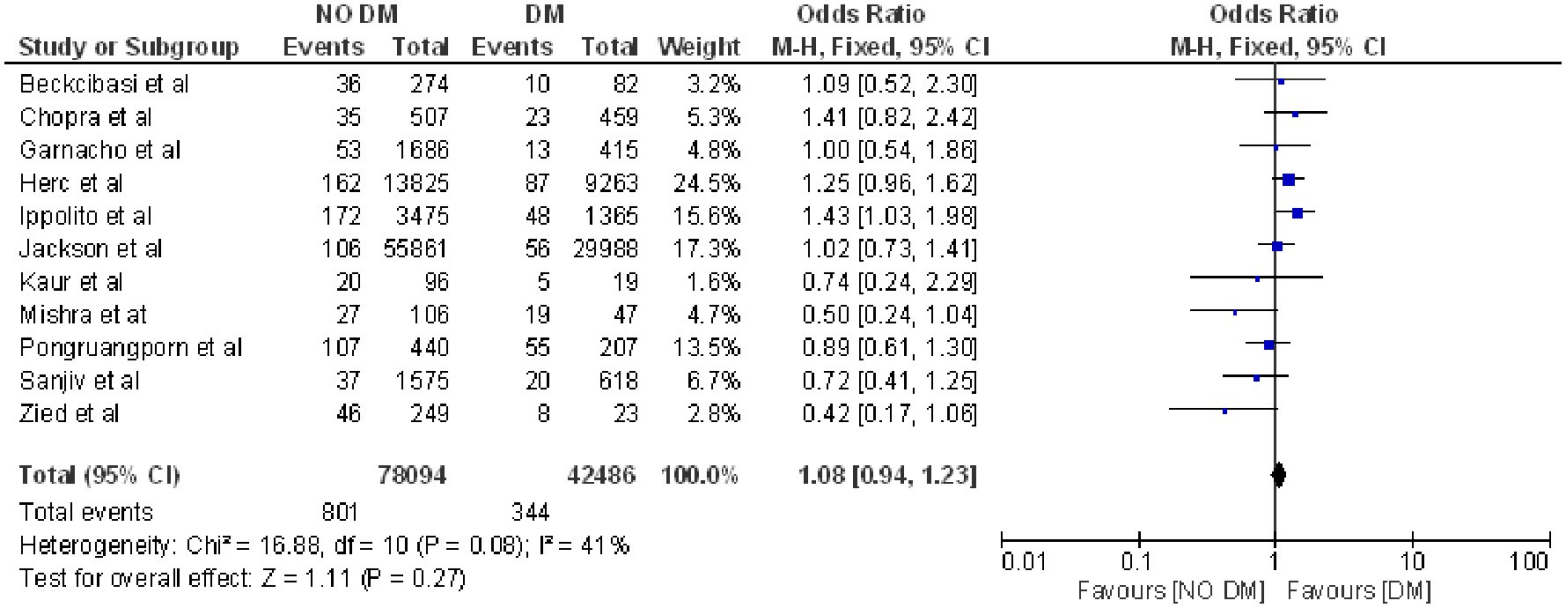
Forest plot of diabetes mellitus and CLABSI.

Likewise, neither was diabetes related to infection [4,17–21, 29, 33, 36, 38, 40] (OR 1.08; 95% CI: 0.94–1.25, p = 0.27 heterogeneity I^2^ = 41%) (Fig 10).

### 3.6 Individual biases

#### 3.6.1

The biases of publication and measurement were cited in 1 or the 23 studied included [31]. The variability in the insertion, management and treatment of CRSBI related to the bias of classification was observed in 3 of the 23 studies evaluated [31, 32, 36]. A bias of detection related to the variability in the definition and measurement of CLABSI was observed in 7 studies [4, 17, 19, 22, 32, 33, 38], and selection bias was detected in 12 studies [4, 16, 18–20, 22, 28–30, 34, 38, 39]. A bias of notification due to missing data during the data collection process was recognized in 12 studies [16, 17, 38, 39, 19–22, 28, 29, 36, 37]. Some studies had a reduced sample size implying a low statistical power in the analysis of some of the risk factors [16, 18, 28, 33]. Finally, 4 studies did not report any limitation [15, 27, 35, 40].

#### 3.6.2

This review has several limitations which are implicit in the studies included in the meta-analysis. Specifically, there was significant heterogeneity in the general results mainly derived from the data belonging to the risk factors of TPN, unilumen and bilumen catheters, days of catheterization, chemotherapy, kidney disease, diabetes and immunosuppression, which were attributed a high-moderate heterogeneity >25%. This heterogeneity could be related to the clinical diversity, sample size and variability of the results since they are very important variables which could explain the heterogeneity of the data as a whole. However, one of the variables studied presented a low heterogeneity < 25% (multilumen catheter) and, thus, may be attributed to very solid results with excellent homogeneity.

## 4. Discussion

The prevention of CLABSI is problematic, with severe clinical repercussions at an individual and organizational level, since the use of venous devices in the hospital setting is a transversal intervention that affects hospitalized, critical, and oncological patients alike. The different studies published show contradictory results and, therefore, the present review has focused on identifying and synthesizing the variables related to the appearance of CLABSI. The results indicate that TPN, multilumen devices, chemotherapy treatment, immune system compromise and the length of catheterization are risk factors for CLABSI. On the other hand, monolumen devices present a lower probability of triggering this infection.

Multiple studies established TPN as a risk factor of CLABSI. The guidelines of the American Society for Parenteral and Enteral Nutrition (ASPEN) and CDC relate TPN with the risk of CLABSI due to the preference of the microorganisms for dextrose [7, 41]. However, ASPEN related other nutritional factors, such as a deficient nutritional status conditioning immune response to the risk of infection. Along the same line, another study corroborated that a state of malnutrition and hypoalbuminemia was associated with CLABSI (OR 3.13; 95% CI 1.38–5.24, p<0.05) [42]. Other studies determined that the risk of CLABSI is dependent on the duration of catheterization and the length of TPN [43, 44]. In addition, it has been shown that manipulation of venous devices and TPN by health care professionals may condition the appearance of CLABSI and should be manipulated with maximum precaution of sterile barriers [7]. Nonetheless, the studies included in this review coincide in establishing TPN as a risk factor, but it should be noted that one study [29] found no association between these two factors, perhaps secondary to the creation of a strategy of bundle manipulation/care/approaches that reduce the appearance of the problem. Therefore, the result of TPN as a risk factor should be interpreted with caution since the factors described could be factors independently related to CLABSI.

Chemotherapy has shown to be an independent factor of CLABSI, but as described in the literature, the cause of this association could be because of the vulnerability of developing any infectious process due to the neutropenia induced by cytostatic drugs [45, 46]. In addition, this study shows that a state of immunosuppression is an independent factor of CLABSI due to immune system dysfunction [13, 47, 48]. However, the studies included in this review did not report whether the cause of the immunosuppression was secondary to a hematological disease, organ transplantation, autoimmune disease or acquired immunodeficiency, and thus, it is not possible to stratify the results based on the causative disease. On the other hand, the results of the meta-analysis identified immunosuppression as an independent risk factor, except in one study due to the reduced sample size [18].

In relation to the number of lumens of the venous devices, multilumen catheters were found to be an individual risk factor of CLABSI. These results coincide with the CDC recommendation (category IB) of implanting devices with the least number of lumens, since the microorganisms reach the catheter through the connections and with these devices the risk is higher due to the greater number of entries [7]. In addition, these devices are susceptible to greater manipulation, hindering adequate disinfection and device maintenance. However, multilumen catheters are indicated in patients with high pharmacologic requirements in whom it is not considered safe to reduce the number of lumens because of the risk of pharmacological interaction [49]. In these cases, the importance of the management and maintenance of these devices is important to note. Along this line, it has been demonstrated that the impregnation of lumens with antimicrobial substances reduces the risk of CLABSI [50].

The present review established that monolumen venous devices are a protective factor; however, a meta-analysis determined that there are no differences when high quality studies with homogeneous samples are analyzed [51]. Therefore, this contradiction among studies could also be related to the quality of management, care and adherence to guidelines by the professionals manipulating these devices [52].

In the case of days of catheterization, the studies included showed elevated heterogeneity in the results. Taking into account that the CDC has established that routine replacement of central devices is not necessary (category IB) [45], it seems that the real reason for the development of infection may be the deterioration and dysfunctionality which venous devices acquire by multiple manipulations over time. Previous studies have shown that the quality of catheter care and management is key in the colonization of these devices [52], with thrombosis and intraluminal and extraluminal fibrin favoring the growth of microorganisms [53].

Infection is the second cause of death in patients with kidney disease receiving hemodialysis therapy [54]. These patients live with precursor risk factors of CLABSI of different causes, such as immune compromise, being carriers of a vascular access for renal replacement therapies, resistance to antibiotics, comorbidities such as diabetes, and colonization by nasal *Staphylococcus aureus* which promote the risk of this infection [55]. However, there are discrepancies among the results obtained in the literature, and our study did not describe any association with catheter-related infection and kidney disease. This may be justified in that the concept of kidney disease is very wide, and all the patients with this disease present very different characteristics which may generate very heterogeneous and inconclusive results. In addition, the CDC states that correct manipulation of a vascular device and correct monitoring by professionals is the main intervention for the prevention of CLABSI [56]. This indicates that depending on the preventive measures applied at an institutional level, having kidney disease is a precursor risk factor for the development of CLABSI.

In our meta-analysis, diabetes was not determined to be an independent risk factor of CLABSI. However, in the literature a relationship has been described between this disease and compromise of immune response [57], which would explain the results of some studies which establish diabetes as a related factor [55]. The discordance of our results with others may be due to the fact that most of the studies included did not take into account the type of diabetes, the complexity of this disease, the treatment or the years of evolution, which could justify the heterogeneity in the results obtained.

In relation to the microbiological results, the most frequent microorganisms isolated were Gram-positive cocci, the most prevalent being coagulase-negative Staphylococci, thereby indicating a possible colonization by skin flora of the patient or secondary to manipulation of the device by different health care professionals. Other series of CLABSI in our setting showed the same trend [58, 59]. However, one study performed in the United States described *Enterobacter* spp. and *Candida* spp. as the most prevalent and concluded that more evidence is necessary to establish why the patients are at risk of presenting CLABSI by these microorganisms to thereby develop preventive measures aimed at these microorganisms [60]. Despite the improvements implemented in recent years, the results demonstrate that studies should be focused not only on strategies of insertion but also on the management and maintenance of venous catheters.

The main limitation of this review is the long interval of time in the inclusion of the articles which may increase the heterogeneity of some of the variables (days of catheterization). Another limitation is that the quality of the studies was good-regular, despite not including any randomized study, and this did not allow the establishment of cause-effect relationship. One other limitation is that the quality of the maintenance of venous devices is a very important factor for the appearance of CLABSI, and its evaluation is difficult to measure and may induce overestimation of the effect of other variables of catheter-related infection. Another aspect to take into account is the elevated heterogeneity based on the variable analyzed. In addition, the large number of variables that can be analyzed as potential related factors are always subject to changes, modifications and extensions of risk factors predisposing to CLABSI, since there are other risk factors not considered in the articles included for analysis that may be related to the appearance of CLABSI. Finally, the last limitation is related to the microbiological results since we were unable to synthetize the results reported in these studies because some are described in real numbers while others are indicated in percentages, and some studies report the species and others the genus.

Despite these limitations, this review also has great strengths such as the meta-analysis which provided a synthesis of the results obtained to date and their clinical applicability.

Robust identification of risk factors may be useful for their inclusion in algorithms for deciding the most adequate venous device, in addition to the variables of pharmacotherapy and venous accesses available. It also allows including therapeutic strategies based on rigorous measures of asepsis with the aim of preventing and reducing the incidence of CLABSI, especially in patients with some of the present risk factors.

## 5. Conclusions

The decision to insert a venous device should be made based on individual evaluation of risk factors for the development of CLABSI since this complication can involve very severe clinical repercussions with very elevated health care costs. Well-designed studies with homogeneous patient samples are needed to increase the quality of the results and help evaluate the efficacy of these devices as well as the clinical benefits and profitability of the therapeutic strategies implemented.

## Supporting information

S1 Text Search strategy. (TIF)

S2 Text PRISMA 2009 checklist. (DOCX)

S3 Text PROSPERO protocol. (PDF)
