# Supplementary figures and images for: Risk factors of catheter- associated bloodstream infection: Systematic review and meta-analysis
Source: PLoS One. 2023 Mar 23;18(3):e0282290. doi: 10.1371/journal.pone.0282290 (PMC10035840; doi:10.1371/journal.pone.0282290)

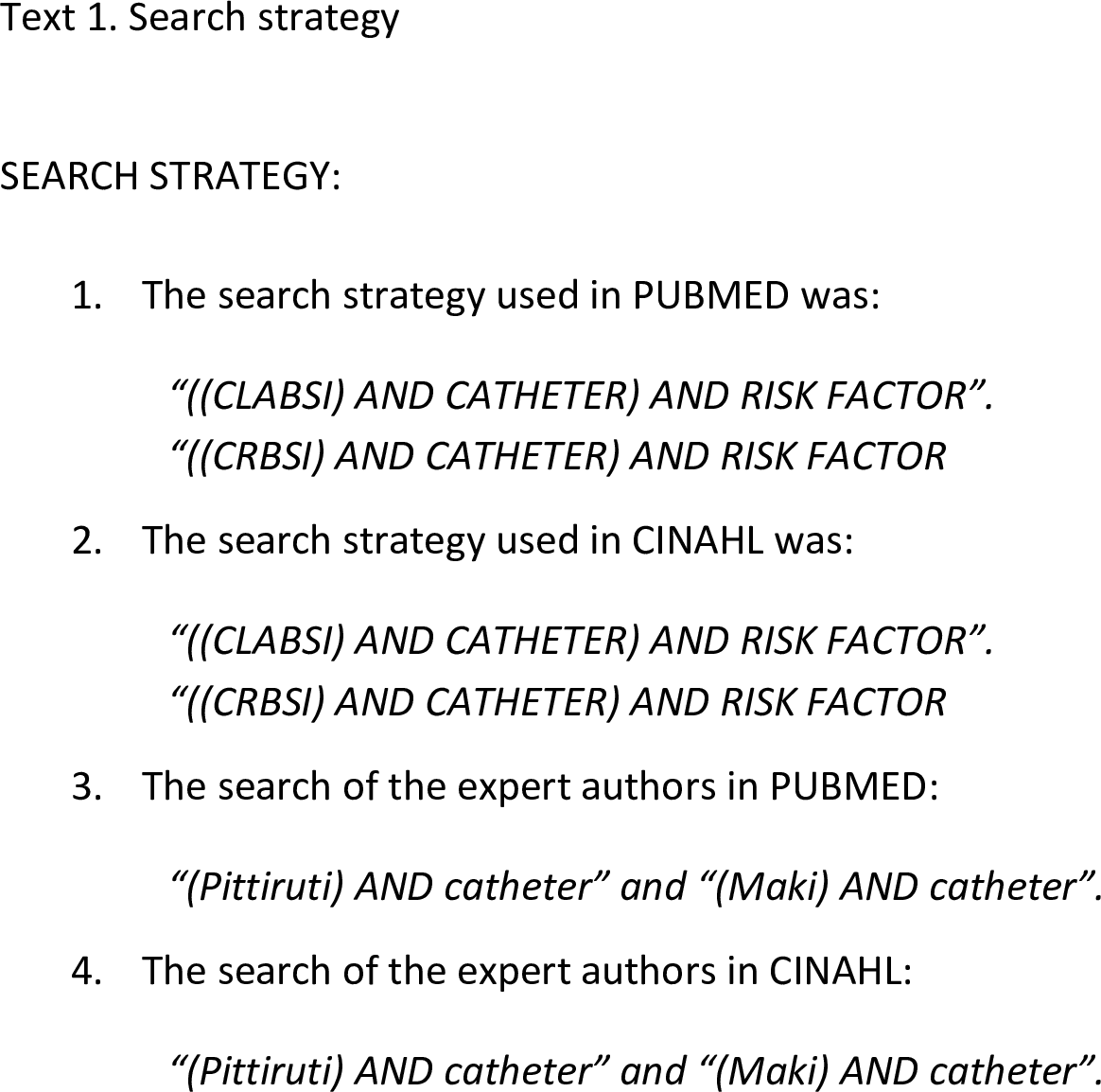

Supplement: S1 Text — (TIF) [file pone.0282290.s001.tif]
